# Overexpression of the Bam Complex Improves the Production of *Chlamydia trachomatis* MOMP in the *E. coli* Outer Membrane

**DOI:** 10.3390/ijms23137393

**Published:** 2022-07-02

**Authors:** Dung T. Huynh, Wouter S. P. Jong, Gregory M. Koningstein, Peter van Ulsen, Joen Luirink

**Affiliations:** 1 Abera Bioscience AB, 2141 Uppsala, Sweden; dung@aberabio.com (D.T.H.); wouter.jong@aberabio.com (W.S.P.J.); 2 Department of Molecular Microbiology, Amsterdam Institute of Molecular and Life Sciences (AIMMS), Vrije Universiteit, 1081 HV Amsterdam, The Netherlands; g.m.koningstein@vu.nl

**Keywords:** *Chlamydia trachomatis* major outer membrane protein, β-barrel assembly machinery, outer membrane protein, *E. coli*, OMV-based vaccine

## Abstract

A licensed *Chlamydia trachomatis (Ct)* vaccine is not yet available. Recombinant *Chlamydia trachomatis* major outer membrane protein (*Ct*-MOMP), the most abundant constituent of the chlamydial outer membrane complex, is considered the most attractive candidate for subunit-based vaccine formulations. Unfortunately, *Ct*-MOMP is difficult to express in its native structure in the *E. coli* outer membrane (OM). Here, by co-expression of the Bam complex, we improved the expression and localization of recombinant *Ct*-MOMP in the *E. coli* OM. Under these conditions, recombinant *Ct*-MOMP appeared to assemble into a β-barrel conformation and express domains at the cell surface indicative of correct folding. The data indicate that limited availability of the Bam complex can be a bottleneck for the production of heterologous OM vaccine antigens, information that is also relevant for strategies aimed at producing recombinant OMV-based vaccines.

## 1. Introduction

*Chlamydia trachomatis* is an obligate intracellular Gram-negative bacterium that is a major cause of common sexually transmitted infection (chlamydia) and blindness (trachoma) worldwide [1,2]. *Chlamydia trachomatis* has a complicated life cycle that begins with metabolically reduced elementary bodies (EBs) that are infectious and invade host cells. After endocytosis, the EBs transform into reticulate bodies (RBs) of low metabolic activity. The RBs then replicate and re-differentiate into the EBs that are released to infect neighboring cells [3,4].

Although treatments exist, chlamydial infections often start asymptomatic, which drives transmission in human populations. When left untreated, an infection can lead to chronic pelvic pain, infertility, and trachoma. The infection can increase the susceptibility to other sexually transmitted pathogens such as HIV, HPV, and Gonorrhea [5]. Therefore, an effective preventive vaccine is considered the best option to reduce chlamydial infection and disease [2,4,6]. A complicating factor in the development of a protective vaccine is the presence of different serotypes and their distinct surface-exposed antigens that correlate with specific immunity. Also, natural infection was shown to provide only transient immunity and the correlates of protection have not been fully elucidated [7]. In any case, both humoral and cellular immune responses appear to be required at the site of infection [8]. Early studies using whole-organism vaccines have been unsuccessful, showing immune responses that were serovar-specific, short-lived, and sometimes even causing delayed hypersensitivity upon re-infection [9]. Although a more recent whole-cell vaccine showed promising results [10], vaccine efforts have shifted towards subunit formulations consisting of protective antigens.

Similar to other Gram-negative pathogens, *Chlamydia trachomatis* outer membrane proteins (OMPs) are considered prime targets for vaccine strategies. Most chlamydial OMPs are rich in cysteine residues that contribute to a cross-linked protein network in the OM to reinforce the OM structure [11,12]. This “chlamydial outer membrane complex (COMC)” is thought to compensate for the absence of a classical circumferential peptidoglycan layer that provides rigidity to the cell envelopes in other Gram-negative species. *Ct*-MOMP (major outer membrane protein) is the best-studied vaccine target and the most abundant constituent of the COMC accounting for as much as 60% of the OM mass [13,14,15]. Due to the presence of both B- and T-cell epitopes, *Ct*-MOMP is strongly immunogenic. It has been proposed to function as a porin in small molecule transport and is predicted to form a classical β-barrel channel lined by amphiphilic antiparallel β-strands [16]. The strands are connected at the surface by flexible extracellular loops that are variable, therefore called VDs (variable domains). The VDs comprise the major B-cell epitopes that underpin the classification of *Chlamydia trachomatis* in serotypes, whereas T-cell epitopes are present in predicted β-strands and periplasmic loops. Native *Ct*-MOMP purified from the EBs induced protective responses against genital infection in mice [12,17]. However, the Ebs are not suitable for large-scale commercial production of *Ct*-MOMP, prompting initiatives for recombinant expression of *Ct*-MOMP in other hosts and exploring the immunogenic properties of non-native *Ct*-MOMP or *Ct*-MOMP domains [18,19]. Encouragingly, a successful clinical phase I trial using a recombinantly engineered *Ct*-MOMP construct was recently reported [20]. This non-native antigen (CTH522) containing VDs of four dominant serovars (D–G), had previously been shown to generate neutralizing antibodies, CD4^+^ T-cell responses, and protective immunity against genital or transcervical challenges in different animal models [21,22,23].

Production of native full-length *Ct*-MOMP in *E. coli* has proven problematic because the expression can affect growth and the protein tends to aggregate, which may be partly due to its unusual cysteine-rich nature [24,25]. In *E. coli*, membrane insertion and folding of β-barrel type OMPs are catalyzed by the Bam (β-barrel assembly machinery) complex in the OM [26,27]. The Bam complex consists of the integral β-barrel-forming subunit BamA that plays a direct role in membrane insertion of nascent OMPs, and four associated lipoproteins, BamB, BamC, BamD, and BamE. The lipoproteins are anchored in the inner leaflet of the OM and fulfill accessory functions in the reception and transfer of nascent OMPs and in the modulation of BamA activity. Recently, we have shown that the availability of the Bam complex in *E. coli* can be a limiting factor in the expression of recombinant autotransporter proteins [28]. Here, we show that co-overexpression of the Bam complex also supports the expression and surface display of *Ct*-MOMP in the *E. coli* OM, which may prove useful for the production of *Ct*-MOMP antigen as such or its incorporation in vaccines based on bacterial OMVs (outer membrane vesicles).

## 2. Results

### 2.1. Expression of Ct-MOMP in E. coli

To express natively folded *Ct*-MOMP in the OM of *E. coli*, a plasmid (pLemo-MOMP) was constructed in which the expression of the *Ct*-MOMP gene from serovar D strain UW3/Cx is under control of the L-rhamnose-inducible *rhaBAD* promoter. This promoter was chosen because it allows for a precise tuning of expression to avoid possible saturation of the membrane translocation channels and chaperones needed for the proper delivery of highly expressed secretory and membrane proteins [29,30,31]. The native signal peptide of *Ct*-MOMP was replaced by an *E. coli* signal peptide (OmpT) for an optimal engagement of the cognate Sec-translocon in the inner membrane [32]. Furthermore, 9 cysteine residues of *Ct*-MOMP were substituted by alanines to prevent potential aggregation resulting from improper intra- and inter-molecular disulfide bond formation by the periplasmic oxidoreductase DsbA (see Supplementary Document 1). The modified *Ct*-MOMP was expressed in *E. coli* BL21(DE3) upon induction with L-rhamnose (2–8 mM) for 2 h (Figure S1). Whole-cell lysates were analyzed by SDS-PAGE and Coomassie staining (Figure S1A) or immunoblotting using *Ct*-MOMP-specific antiserum for detection (Figure S1B). Tightly regulated expression of *Ct*-MOMP at its expected molecular weight (~41 kDa) was observed by immunoblotting, with increasing L-rhamnose concentrations leading to increasing levels of *Ct*-MOMP (Figure S1B). However, even at the highest level of induction, *Ct*-MOMP could not be clearly distinguished by Coomassie staining (Figure S1A, lane 4).

### 2.2. Improvement of Ct-MOMP Levels upon Co-Overexpression of the E. coli Bam Complex

**Figure 1:**
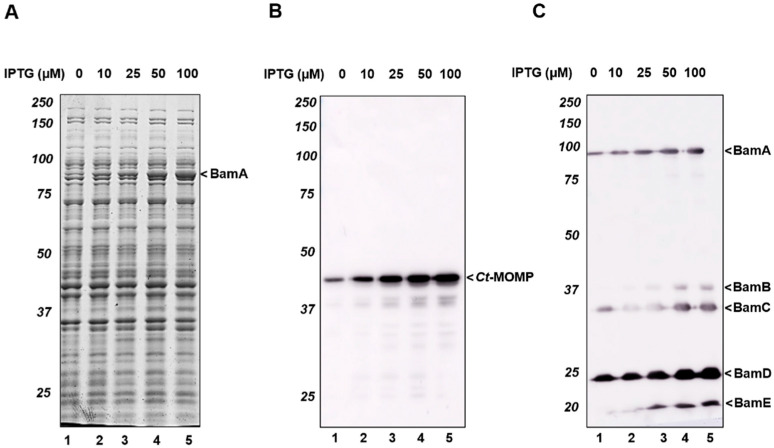
Co-expression of the Bam complex improved the production of *Ct*-MOMP in *E. coli*. (**A**) *E. coli* BL21(DE3) cells co-expressing *Ct*-MOMP and *E. coli* Bam were analyzed by SDS-PAGE and Coomassie staining. Cells were induced for expression of the Bam complex with various concentrations of IPTG (0–100 µM) for 1 h and subsequently induced for production of *Ct*-MOMP with 8 mM L-rhamnose for 2 h. (**B**,**C**) Immunoblotting analysis of the whole-cell lysates using antisera against *Ct*-MOMP (**B**) or the Bam complex (**C**). Molecular weight (kDa) markers were indicated at the left side of the panels, the identified protein bands were indicated at the right side of the panels.

We then considered the possibility that the observed low expression of *Ct*-MOMP in *E. coli* was due to inefficient insertion into the OM provoking recognition and degradation by the periplasmic quality control systems [33]. Potentially, this may originate from a mismatch between the heterologous *Ct*-MOMP and the *E. coli* Bam complex. Recently, we have shown that co-overexpression of the *E. coli* Bam complex improves the secretion of difficult-to-secrete recombinant autotransporter chimeras that include an OM-inserted β-barrel domain and are sensitive to degradation when accumulating in the periplasm [28]. To test whether this approach could also relieve a bottleneck in the production of *Ct*-MOMP, we co-transformed cells carrying pLemo-MOMP with a compatible plasmid, pJH114, that encodes all subunits of the *E. coli* Bam complex under control of the IPTG-inducible *trc* promoter [34]. To ensure the availability of additional Bam complexes for *Ct*-MOMP biogenesis, production of the Bam subunits was induced 1 h prior to induction of *Ct*-MOMP expression (with 8 mM L-rhamnose). Cell samples were subsequently taken after 2 h and analyzed by SDS-PAGE and Coomassie staining (Figure 1A) or immunoblotting using antisera against either *Ct*-MOMP or the Bam complex (Figure 1B,C, respectively). As expected, gradually increasing levels of Bam subunits accumulated in the cells (Figure 1C) in an IPTG-concentration dependent manner. BamA could be readily detected upon Coomassie staining, whereas detection of other Bam subunits (BamBCDE) appeared obscured by endogenous proteins (Figure 1A). Importantly, improved expression of *Ct*-MOMP was observed by immunoblotting (Figure 1B) that resulted from increasing the Bam complex levels (Figure 1C). This suggests that co-overexpression of the Bam complex has a favorable impact on the biogenesis of *Ct*-MOMP in *E. coli.* A regime involving induction of Bam expression with 100 μM IPTG 1 h prior to the addition of 8 mM L-rhamnose yielded the highest levels of *Ct*-MOMP and was selected for our further studies on *Ct*-MOMP biogenesis (Figure 1B and Figure S3).

To exclude that the observed effects were strain-specific for *E. coli* BL21(DE3), we tested the influence of Bam overexpression on *Ct*-MOMP biogenesis in different *E. coli* and *Salmonella* Typhimurium backgrounds. *E. coli* DH5α was chosen as representative of the *E. coli* K12 lineage, while BL21(DE3)-derivative ClearColi(DE3) [35] and *Salmonella* Typhimurium SL3261-∆*msbB* [36] were selected because these strains express detoxified versions of lipopolysaccharide, which is relevant for bacteria-based vaccine development. Induction of the Bam complex from pJH114 with IPTG resulted in a Coomassie-detectable increase in BamA levels (Figure S2, lanes 2, 4, 6). Importantly, *Ct*-MOMP levels were also found to increase in these cells (Figure S2A, lane 2, 4, 6) compared to cells not induced with IPTG (Figure S2B, lane 1, 3, 5), which suggests that the favorable impact of Bam overexpression on *Ct*-MOMP biogenesis is not specific and can be achieved in relevant vaccine strains.

### 2.3. The Complete Bam Complex Is Needed for Optimal Ct-MOMP Expression

**Figure 2:**
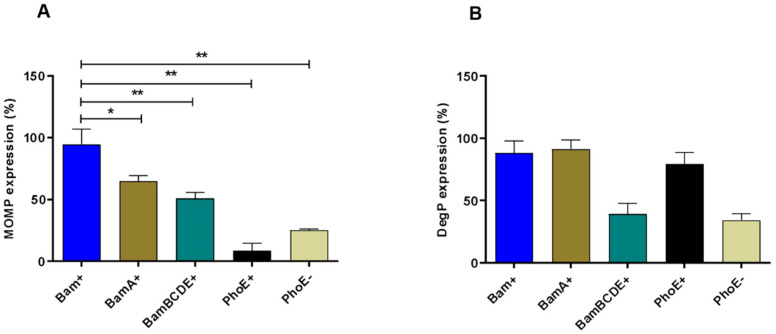
Comparison of *Ct*-MOMP expression in the presence of the full Bam complex, BamA, or BamBCDE subcomplex. Cells co-expressing *Ct*-MOMP and either the Bam complex (Bam+), BamA, BamBCDE, or PhoE were analyzed by immunoblotting using antisera against *Ct*-MOMP, DegP, and RpoB. Quantification of signals from samples displayed in Figure S5, Figure S6 was shown. *Ct*-MOMP (**A**) and DegP (**B**) expression levels in cells co-expressing BamA, BamBCDE, and PhoE were calculated relative to those of cells co-expressing the Bam complex (Bam+). Quantification of *Ct*-MOMP and DegP levels was normalized for quantification of RpoB levels in the same sample. Measurements were based on three independent experiments. Statistical differences were calculated using Student’s *t*-test for unpaired means. “*”: *p* < 0.05; “**”: *p* < 0.01.

Within the Bam complex, the BamA subunit has a central role in folding and integrating OMPs into the OM lipid bilayer [37], whereas BamBCDE subunits have regulatory functions [38]. To investigate which of these roles are most relevant for improving *Ct*-MOMP biogenesis, we monitored cellular *Ct*-MOMP levels upon overexpression of either the BamA or BamBCDE subunits and compared these to cells overexpressing Bam units in three independent experiments. As a negative control to the Bam complex, the unrelated β-barrel OMP PhoE was expressed together with *Ct*-MOMP. IPTG-induced expression of BamA, BamBCDE, or Bam subunits, as well as PhoE was confirmed by analysing whole-cell samples by SDS-PAGE and Coomassie staining (Figure S4). Notably, the levels of BamA appeared somewhat lower when expressed by itself (e.g., Figure S4, cf lanes 2 and 4), which may relate to the stabilizing effect of BamD on BamA [38]. Semi-quantitative immunoblotting on whole-cell samples (Figure S5) was performed to compare *Ct*-MOMP expression levels in the different co-expression conditions (Figure 2A). Levels of *Ct*-MOMP were normalized against those of RpoB (DNA-directed RNA polymerase subunit β), a cytosolic protein that is expected to be insensitive to variations in Bam expression (Figure S5, see Supplementary Document 2). In line with the visually observed differences, a considerable ~4-fold improvement of *Ct*-MOMP expression was found upon co-expression of the full Bam complex compared to cells only induced for *Ct*-MOMP (Figure 2A, compare Bam+ to PhoE−). Co-expression of only BamA also resulted in significantly higher levels of *Ct*-MOMP but to a lesser extent compared to the effect of full Bam complex co-expression. The difference could be due to the lower expression of the orphan BamA subunit as discussed above. Co-expression of BamBCDE had a more moderate effect, despite the efficient expression of these subunits in the absence of BamA (Figure 2A, compare BamBCDE+ to Bam+). In any case, the combined data indicate that both the accessory subunits and BamA contribute to *Ct*-MOMP expression, but the complete Bam complex yields the most pronounced effect.

Overexpression of periplasmic proteins and OMPs may induce cell envelope stress [39], and may have resulted in the moderately decreased growth observed for cultures induced for Bam and MOMP (Figure S3). To test whether Bam overexpression induces cell envelope stress, we monitored its effect on the production of the chaperone/protease DegP, which is upregulated under several envelope stress conditions [33]. Indeed, a clear IPTG-dependent increase of DegP expression was observed in pJH114-containing cells (Figure S3). This prompted us to rule out that enhanced *Ct*-MOMP production is a consequence of secondary stress-related effects rather than of Bam overexpression itself. Overexpression of the unrelated OM protein PhoE also induced upregulation of DegP, similar to BamA and the complete Bam complex (Figure 2B, Figures S4 and S6), but did not promote expression of *Ct*-MOMP (Figure 2A, Figures S4 and S5). In fact, *Ct*-MOMP expression was even lower than in cells not induced for PhoE expression (Figure 2A, compare PhoE+ to PhoE−), which could be caused by DegP-mediated degradation of *Ct*-MOMP intermediates accumulating in the periplasm (Figure 2B, compare PhoE+ to PhoE−). Taken together, the data support a scenario in which co-overexpression Bam directly improves the expression of *Ct*-MOMP.

### 2.4. Localization of Ct-MOMP in the OM

**Figure 3:**
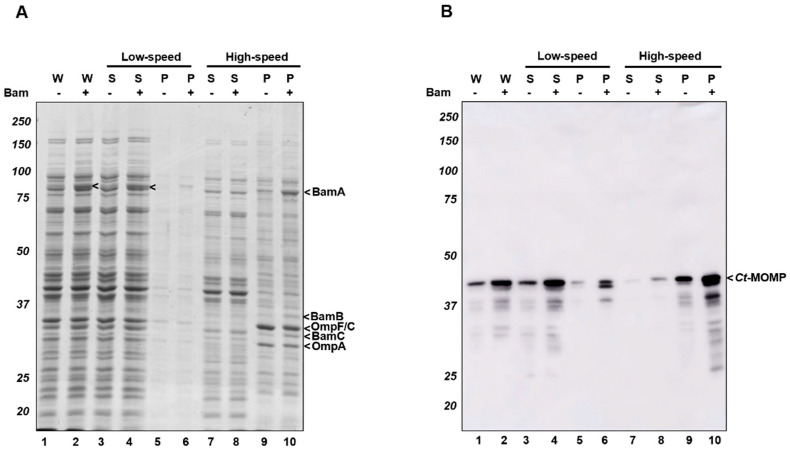
*Ct*-MOMP localized in the *E. coli* cell envelopes. Subcellular fractionation of cells co-expressing *Ct*-MOMP in the presence or absence of the Bam complex. Cells were induced (+) (100 µM IPTG, 1 h) or not induced (−) for expression of the Bam complex followed by induction for expression of *Ct*-MOMP (8 mM L-rhamnose, 2 h). Samples derived from whole-cell lysates (W) and pellets (P) and supernatants (S) produced by subsequent low-speed and high-speed centrifugation steps were analyzed by SDS-PAGE/Coomassie staining (**A**) or immunoblotting using *Ct*-MOMP antiserum (**B**).

To examine whether recombinant *Ct*-MOMP was properly delivered in the OM, we subjected *E. coli* cultures expressing *Ct*-MOMP to subcellular fractionation. Initially, we tested whether the protein is present in crude cell envelopes, comprising both the bacterial inner and outer membranes (Figure 3). Cells expressing *Ct*-MOMP either in the absence or presence of induced Bam complex were lysed and subjected to differential centrifugation followed by analysis of the fractions by SDS-PAGE and Coomassie staining (Figure 3A). The major OMP OmpF/C and OmpA were almost exclusively detected in the cell membranes (Figure 3A; compare lanes 9–10 to lanes 7–8), validating the fractionation procedure. Likewise, although BamD and BamE were difficult to detect by Coomassie staining, BamA, -B, and -C subunits localized to the cell envelopes (Figure 3A, lanes 9–10), indicating a proper localization of the Bam components even when overexpressed, consistent with the earlier studies [40,41]. *Ct*-MOMP localization was monitored by immunoblotting (Figure 3B). The protein did not appear to be aggregation-prone as it was predominantly recovered in the low-speed supernatant, irrespective of co-expression of the Bam complex (Figure 3B, lanes 3–4). Upon subsequent high-speed centrifugation, *Ct*-MOMP was almost exclusively detected in the pellet fraction indicative of membrane association (Figure 3B, lanes 9–10).

**Figure 4:**
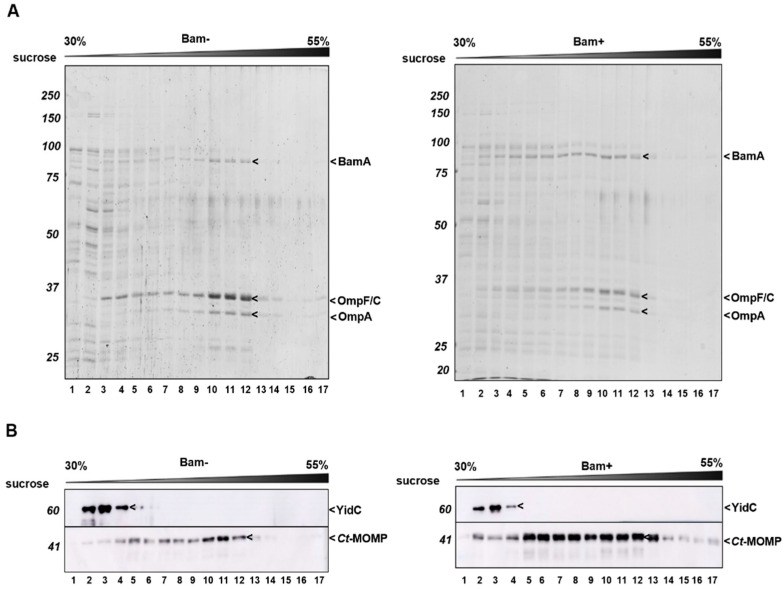
*Ct*-MOMP localized to the OM. Isopycnic sucrose gradient centrifugation of cell envelopes derived from *E. coli* co-expressing *Ct*-MOMP in the absence (−) or presence (+) of the Bam complex. Cell envelopes were derived from the high-speed pellets described in the legend in Figure 3. Fractions (1–17) collected from the top to bottom of each tube were analyzed by SDS-PAGE/Coomassie staining (**A**) or immunoblotting using antisera against *Ct*-MOMP or YidC as indicated (**B**).

Next, we wished to verify the localization of *Ct*-MOMP in the OM. Cell envelopes isolated from cells co-expressing *Ct*-MOMP with (Bam+) or without (Bam−) the Bam complex were subjected to isopycnic sucrose gradient centrifugation to separate the inner and outer membranes [42] (Figure 4). Analysis of the fractions by SDS-PAGE and Coomassie staining showed that OM marker proteins OmpF/C and OmpA peaked in fractions 10–12 irrespective of co-expression of the Bam complex (Figure 4A). Immunoblotting of the fractions further demonstrated that the integral inner membrane protein YidC was mostly present in fractions 2–4 (Figure 4B). As expected, endogenous BamA peaked in fractions 10–12 together with OmpF/C and OmpA (Figure 4A and Figure S7). Induced *Ct*-MOMP from cells not induced for Bam overexpression also peaked in fractions 10–12, suggesting localization with BamA in the OM (Figure 4A). Though distributed more broadly over the gradient, the increased levels of *Ct*-MOMP in cells co-overexpressing Bam also largely co-fractionated with BamA with a slight peak in fractions 10–12 (Figure 4B). Importantly, it was hardly detected in the fractions containing the inner membrane marker YidC (Figure 4B, fractions 2–4). Virtually no *Ct*-MOMP was found at the bottom of the gradient (Figure 4B, fraction 17), confirming that the recombinant protein did not form large aggregated structures. In conclusion, the recombinant *Ct*-MOMP predominantly localized in the OM regardless of Bam co-overexpression.

**Figure 5:**
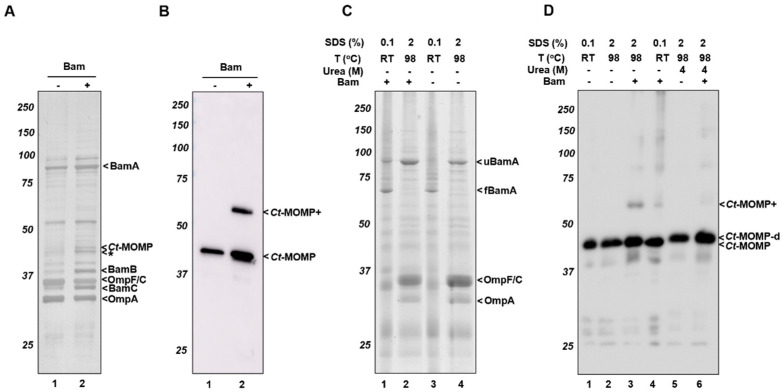
Localization and heat-modifiability of *Ct*-MOMP in the *E. coli* OM. Sucrose density gradient fractions 10–12 derived from cells expressing *Ct*-MOMP either in the absence (−) or presence (+) of the Bam complex (see Figure 4) were pooled and subjected to ultracentrifugation to collect the OMs. After washing, the membranes were analyzed by SDS-PAGE and silver staining (**A**) or immunoblotting using *Ct*-MOMP antiserum (**B**). The same samples were used for analyzing the heat-modifiability of *Ct*-MOMP. Membranes were solubilized in the presence of 0.1% (*w*/*v*) SDS to obtain semi-native samples or 2% (*w*/*v*) with or without 4 M urea to obtain denatured samples. Samples were kept at room temperature (RT) or heated (98 °C) prior to analysis by semi-native SDS-PAGE followed by Coomassie staining (**C**) and immunoblotting (**D**). A silver-stained band at ~41 kDa representing *Ct*-MOMP that could be identified through immunoblotting was indicated (*Ct*-MOMP). A similar-sized silver-stained product that was not detected by *Ct*-MOMP antiserum (*) and the ~60–65 kDa band potentially representing a higher-order form of *Ct*-MOMP was also annotated (*Ct*-MOMP+). In panel C, bands representing folded BamA (fBamA) and denatured unfolded BamA (uBamA) were indicated.

To analyze the protein content of OM fractions isolated from cells overexpressing or not overexpressing the Bam complex (Bam+ and Bam-, respectively) in more detail, the membranes from fractions 10–12 of the respective sucrose gradients were pooled, collected, and reanalyzed by SDS-PAGE and silver staining (Figure 5A). While similar levels of OmpF/C and OmpA were detected in both samples, elevated levels of BamA, -B and -C were clearly detected in the Bam+ samples. Interestingly, in the ~41 kDa size range, novel protein bands emerged in the Bam+ sample (Figure 5A, lane 2). The identity of the lower band (*) remains unclear, but immunoblotting confirmed that the upper band represented *Ct*-MOMP (Figure 5B, lane 2). Remarkably, immunoblotting detection using *Ct*-MOMP antiserum revealed a second band at ~60–65 kDa. This band may represent an SDS-resistant multimeric form of *Ct*-MOMP, which also has been observed for recombinant *Ct*-MOMP produced in nanodiscs [43]. Moreover, a protein product with very similar electrophoretic behaviour was described to represent a compact trimeric form of native *Ct*-MOMP when purified from EBs [16]. In any case, the data demonstrate the production of a substantial, silver stain-detectable amount of *Ct*-MOMP in the *E. coli* OM, but only upon co-expression of the Bam complex.

### 2.5. Heat-Modifiability of Ct-MOMP

In general, the folding state of a β-barrel membrane protein can be assessed using semi-native SDS-PAGE [44,45]. A β-barrel OMP usually remains folded even in the presence of low concentrations of SDS, provided that the samples are not heated. This results in an altered, usually faster, migration of the folded protein in the gel when compared to a heated, fully denatured sample. For native *Ct*-MOMP such a heat-modifiability assay previously resulted in a more complicated pattern. Bands at ~41 kDa represented stable and denatured forms of *Ct*-MOMP monomer while a ~60–65 kDa band was likely to represent stable trimers with also less-stable multimers running higher position in the gel [16]. To test the heat-modifiability of recombinant *Ct*-MOMP expressed in *E. coli* BL21(DE3), we incubated the pooled OM fractions from the sucrose gradients in different loading buffers (0.1% SDS or 2% SDS). All samples were kept at RT or heated (98 °C) prior to loading on semi-native SDS-PAGE. The gels were either stained with Coomassie or subjected to immunoblotting to detect *Ct*-MOMP (Figure 5C,D). The Coomassie-stained gels showed that BamA in the OM fractions was clearly heat-modifiable, (Figure 5C, lanes 1–4), with folded forms of BamA (fBamA) disappearing upon heat treatment in favour of unfolded forms (uBamA), similar to the previous reports [46,47]. In all samples, regardless of whether the Bam complex was overexpressed or not, a major *Ct*-MOMP band running at ~41 kDa did not shift upon heating of the 2% SDS samples (Figure 5D, lanes 1–4). Additionally, in the heated OM sample of Bam+ cells, the band of ~60–65 kDa appeared (Figure 5D, lane 3), possibly representing the stable trimer revealed previously for native *Ct*-MOMP [16]. In the non-heated OM sample with 0.1% SDS, this band was much less prominent, perhaps because it resolved into higher-order complexes detected as a smear high up in the gel (Figure 5D, lane 4), consistent with the previous study [43].

In an attempt to fully denature the ~60–65 kDa band, we incubated the OM fractions in loading buffer containing both 2% SDS and 4 M of urea, then heated these samples (Figure 5D, lanes 5–6). As expected, the addition of urea resulted in the disappearance of the *Ct*-MOMP+ band. Strikingly, heating in 4 M urea also changed the migration of the monomeric *Ct*-MOMP bands to a slightly higher position (Figure 5D, compare lanes 5–6 to lanes 1–4), which could reflect further denaturation of the monomeric form also observed for native *Ct*-MOMP [16].

### 2.6. Ct-MOMP Is Exposed at the Surface of E. coli

**Figure 6:**
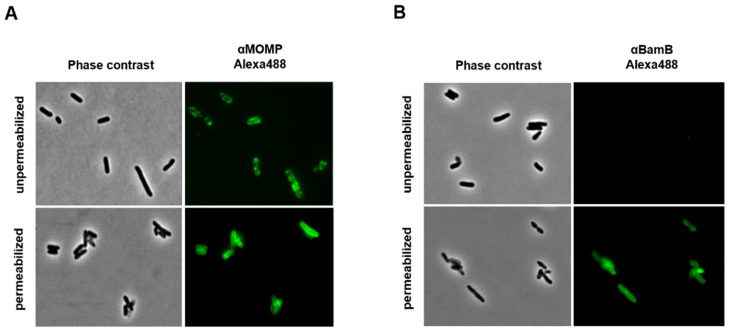
*Ct*-MOMP was exposed at the surface of *E. coli*. Cells co-expressing *Ct*-MOMP and Bam complex were fixed and subjected to indirect immunofluorescence microscopy using rabbit polyclonal antisera against *Ct*-MOMP (**A**) or BamB (**B**) and Alexa Fluor 488 labeling/conjugation of antiserum. The intact cells were permeabilized with Triton-X100 and lysozyme prior to antisera incubations when indicated. Corresponding phase-contrast microscopy fields were also shown. The scale bar was 10 µm. Images were processed using ImageJ software (http://rsb.info.nih.gov/ij/).

When fully integrated into the OM, *Ct*-MOMP is expected to expose several loops to the extracellular milieu [48]. To assess surface exposure of heterologous *Ct*-MOMP in *E. coli* BL21(DE3) co-overexpressing *Ct*-MOMP and the Bam complex, cells were subjected to immunofluorescence microscopy using *Ct*-MOMP-specific antiserum (Figure 6A). A clear circumferential, punctuate labeling of intact cells was observed, indicating *Ct*-MOMP indeed exposed domains at the cell surface. Interestingly, similar to *Ct*-MOMP, the Bam complex has been described to localize in bright distinct foci in immunofluorescence studies [49,50]. Permeabilization of the cells with Triton X-100 and lysozyme prior to the antiserum incubation yielded a more homogenous circumferential labeling, which could indicate improved access of the antiserum to also intracellular parts of *Ct*-MOMP. As a further control for cell integrity, we performed labeling of lipoprotein BamB, which is located on the periplasmic side of the OM and thus not accessible to antisera when cells are intact. Indeed, BamB could not be detected with BamB antiserum, unless cells were first permeabilized (Figure 6B). In conclusion, we demonstrated that *Ct*-MOMP inserted into the *E. coli* OM exposes domains at the cell surface.

## 3. Discussion

The abundance and immunogenicity of *Ct*-MOMP make it the most attractive *Chlamydia* vaccine target despite its moderate conservation and despite the challenges to express and purify sufficient amounts of properly folded *Ct*-MOMP from native or heterologous hosts. Here, we show that the expression and localization of recombinant *Ct*-MOMP in the *E. coli* OM can be substantially improved by co-overexpression of the Bam complex, suggesting that Bam-mediated assembly of *Ct*-MOMP is a bottleneck in its biogenesis.

Expression of recombinant *Ct*-MOMP in *E. coli* has shown to be problematic [32,51], which may be due to the intricate species-specific conformation of native *Ct*-MOMP. Its three-dimensional structure has not been solved to date, but sequence-based predictions suggested it folds into a 16-stranded β-barrel [15,52]. These models placed the immunogenic VDs of *Ct*-MOMP at the cell surface. We have used the recent AlphaFold protein structure prediction method [53] to model the native *Ct*-MOMP. Interestingly, and in contrast to the previous predictions, it yielded a structural model of high confidence that includes only 10 very long antiparallel β-strands, while still placing all VDs at the cell surface (Figure S8A). Possibly, the extended β-barrel structure is related to the relatively long fatty acid chains of *Chlamydia trachomatis* lipid A, when compared to other Gram-negative bacteria [54]. This creates a relatively thick hydrophobic core in the OM, which may impact the nature of transmembrane anchoring and explain the longer β-strands. If correct, this is also expected to complicate the Bam-mediated assembly of heterologous *Ct*-MOMP as it is thought to involve an intermediate “hybrid-barrel” folding state [55]. The formation of a poorly matched interface of the nascent *Ct*-MOMP β-strands and the short β-strands that flank the lateral gate of the “insertase” BamA in *E. coli* may potentially slow down *Ct*-MOMP membrane integration. This, in turn, could induce cell-envelope quality control mechanisms, as suggested by the increased levels of DegP detected, triggering degradation of nascent *Ct*-MOMP. Overproduction of the Bam complex may overcome titration of available Bam complexes handling recombinant *Ct*-MOMP resulting in improved assembly rates. A similar beneficial effect of the Bam complex overproduction was recently reported for the surface display of difficult-to-secrete chimeric autotransporter constructs that also obstruct the Bam complex [28].

An additional problem in recombinant expression may be posed by the 9 cysteines in *Ct*-MOMP, an unusually high number for an OMP. In *Chlamydia trachomatis* they form intermolecular disulfide bonds that contribute to a supportive connected network of OMPs in the cell envelopes know as the COMC [56]. However, in the absence of cognate partner proteins and in the presence of a non-cognate oxidoreductase system, non-native disulfide bonding of these cysteines may lead to misfolding and consequent degradation in the *E. coli* periplasm. The substitution of the cysteines for alanines has been shown to improve *Ct*-MOMP production to some extent [32], but our results, obtained with a cysteineless variant, indicate that the OM integration is still a bottleneck for this variant. Of note, an AlphaFold model for this variant closely resembles that of non-mutated *Ct*-MOMP (Figure S8B). If the model is correct, the relatively long antiparallel β-strands may also explain the harsh conditions that appear required for full denaturation of the integrated *Ct*-MOMP.

It is difficult to compare the extent of *Ct*-MOMP expression, localization, folding, and surface display presented here with other attempts to improve recombinant *Ct*-MOMP expression as different assays for detection have been applied. In general, expression of recombinant *Ct*-MOMP has benefited from the use of an *E. coli* signal peptide [32], tailored growth conditions, optimal tuning of protein expression by codon harmonization [57] and the use of well-regulatable promoters such as the L-rhamnose-inducible *rhaBAD* promoter applied in this study. Conceivably, the gradual expression of *Ct*-MOMP leads to an optimal use of the Sec-translocon, which is known to be a potential bottleneck for membrane protein production [58]. Alternative reported approaches to increase production of *Ct*-MOMP antigen include cell-free expression in nanolipoprotein particles [43] and grafting VDs of *Ct*-MOMP in another β-barrel OMP, PorB of *Neisseria lactamica* [17,18]. Parallel efforts are directed towards the high-level expression of non-native *Ct*-MOMP (lacking a signal peptide) that can be relatively easily produced in the cytosol of *E. coli*. For example, the engineered version of *Ct*-MOMP (CTH522) that comprises VDs of different serovars may lack conformational B-cell epitopes but still generates neutralizing antibodies and cellular immune responses [59,60].

## 4. Conclusions

In conclusion, co-overexpression of the Bam complex is a promising strategy to increase the expression of challenging heterologous OMPs in the *E. coli* OM. This may prove useful for the purification of native OMP antigens for vaccine development or for structural and functional analysis. Furthermore, the inclusion of substantial amounts of native antigens in the OM may enable the generation of recombinant live or OMV-based vaccines. From that perspective, it is encouraging that *Ct*-MOMP expression in an attenuated *Salmonella* Typhimurium strain suitable for vaccine production can be achieved using this strategy. Future efforts will be directed towards the development of OMV-based vaccines that include *Ct*-MOMP and preferably also other *Chlamydia trachomatis* OMPs.

## 5. Materials and Methods

### 5.1. Bacterial Strains and Growth Media

*E. coli* DH5α (Novagen, Darmstadt, Germany) was used for cloning. *E. coli* BL21(DE3), Clearcoli(DE3) (Novagen, Darmstadt, Germany) and *Salmonella enterica* serovar Typhimurium SL3261-∆*msbB* strains were applied for protein expression experiments. The latter strain was created by allelic exchange through double cross-over homologous recombination as described [61] using *S*. Typhimurium SL3261 [62] as the parental strain. DH5α and BL21(DE3) were cultured in lysogeny broth (LB; 10 g/L bacto tryptone, 5 g/L bacto yeast extract, 10 g/L NaCl) containing 0.2% glycerol at 37 °C. Clearcoli(DE3) was grown in the same medium supplemented with 1 mM MgSO_4_ SL3261-∆*msbB* was cultured in TYMC (10 g/L tryptone, 5 g/L yeast extract, 2 mM MgSO_4_, 2 mM CaCl_2_) containing 0.2% glycerol at 30 °C. Chloramphenicol (30 μg/mL) and ampicillin (100 μg/mL) antibiotics were added for plasmid maintenance, when required.

### 5.2. Reagents, Enzymes, and Sera

Plasmid DNA Miniprep and MACHEREY-NAGEL NucleoSpin Gel, PCR Clean-up gel extraction kits were obtained from Thermo Fisher Scientific (Waltham, MA, USA). Phusion High Fidelity DNA polymerase and DNA restriction enzymes were bought from New England Biolabs (Ipswich, MA, USA). In-Fusion HD Plus Kit for seamless DNA cloning was obtained from Takara Bio (Mountain View, CA, USA). Coomassie Blue G250 was from BioRad (Hercules, CA, USA). Silver staining kit was obtained from Thermo Fisher Scientific (Waltham, MA, USA). Lumi-Light substrate for immunoblotting was purchased from Roche (Mannheim, Germany). All other reagents and chemicals were supplied by Sigma–Aldrich (Saint Louis, MO, USA).

Rabbit antiserum against *Ct*-MOMP was kindly provided by Frank Follmann (Statens Serum Institut, Copenhagen, Denmark). Rabbit antiserum against DegP was a gift of Jon Beckwith, Harvard Medical School, USA. The YidC polyclonal antiserum was raised in rabbits against a peptide that consisted of the 17 C-terminal amino acids of YidC by Agrisera (Umea, Sweden) [63]. Mouse antiserum against *E. coli* RpoB was bought from BioLegend (San Diego, CA, USA). Rabbit antisera against Bam, BamA and BamB were provided by Tanneke den Blaauwen (University of Amsterdam, Amsterdam, The Netherlands). Alexa488 goat anti-rabbit IgG was obtained from Life Technologies (Willow Creek Rd, Eugene). Anti-mouse and anti-rabbit peroxidase IgG antisera generated in goats were ordered from Rockland (Limerick, PA, USA).

### 5.3. Plasmid Constructions

An *E. coli* codon-optimized synthetic DNA fragment (1180 bp) encoding the mature MOMP of *Chlamydia trachomatis* serovar D (UW3/Cx) (UniProtKB-Q9RB77) fused to the *E. coli* OmpT signal peptide was ordered from Invitrogen Geneart (Waltham, MA, USA). The 9 cysteines of *Ct*-MOMP were replaced by alanines (see Supplementary Document 1). The OmpT-MOMP DNA was amplified by PCR using primers 1–2 (see Supplementary Document 1—Table S1). To place the *Ct*-MOMP-encoding sequence under control of the L-rhamnose-inducible *rhaBAD* promoter, the PCR product was ligated into the *EcoR*I/*Nde*I sites of pLemo-HbpD-H56(dTM) [28] using In-Fusion cloning, resulting in pLemo-MOMP.

The pJH114 plasmid [34] encoding the ABCDE subunits of the Bam complex under control of the IPTG-inducible *trc* promoter was kindly provided by Harris Bernstein (Bethesda, USA). To generate versions of pJH114 expressing either the BamA or BamBCDE subunits, BamA and BamBCDE encoding DNA sequences were amplified by PCR using pJH114 as the template and primers 3–4 and primers 5–6, respectively (see Supplementary Document 1—Table S1). PCR fragments were ligated into *Nde*I/*Xba*I-digested pJH114 using In-Fusion cloning so as to yield pTrc-BamA and pTrc-BamBCDE. A DNA sequence encoding *E. coli* K12 PhoE was cloned under *lacUV5* promoter control into vector pEH1 [64], yielding plasmid pEH1-PhoE. All constructs were confirmed by sequencing (Macrogen, Amsterdam, The Netherlands).

### 5.4. General Protein Production and Analysis

*E. coli* BL21(DE3) cells harboring pLemo-MOMP were cultured until an OD_600_ of 0.4–0.5, at which time expression of *Ct*-MOMP was induced by adding 0, 2, 4, 8 mM L-rhamnose and growth was continued for 2 h. For co-expression, cells harboring pLemo-MOMP and any of pJH114, pTrc-BamA, pTrc-BamBCDE or pEH1-PhoE, were induced with 100 µM IPTG at an OD_600_ of 0.4–0.5 for production of the Bam complex, Bam subunits (BamA or BamBCDE) or PhoE (negative control), respectively. Cultures were grown for 1 h, after which expression of *Ct*-MOMP was induced by adding L-rhamnose to a final concentration of 8 mM, and further grown for 2 h. Then, cells and culture media were separated by centrifugation (10,000× *g,* 10 min, 4 °C). Cell pellets were resuspended in an ice-cold lysis buffer (5 mM Tris-HCl pH 7.4, 100 mM NaCl, 1 mM EDTA). Next, the cells were disrupted by tip sonication (Branson Sonifier 250), yielding whole-cell lysates. Whole-cell lysates were analysed by SDS-PAGE and Coomassie Blue G250/silver staining or immunoblotting. Densitometric scanning on Coomassie-stained or silver-stained gels was conducted using a Molecular Imager GS800 Calibrated Densitometer from Life Science (Bulletin, USA). Immunoblots were imaged using an AI600 Imager from GE Healthcare (Canton, MA, USA).

### 5.5. Subcellular Fractionation

The cell pellets were resuspended in a lysis buffer (5 mM Tris-HCl pH 7.4, 100 mM NaCl, 1 mM EDTA) plus the complete protease inhibitor from Roche (Mannheim, Germany) to a working concentration (a tablet per 50 mL extraction solution). Cell suspensions were kept in ice for 15 min. Cells were broken by two passages through the One-Shot disruptor from Constant System Ltd. (Daventry, UK) at 1.9 kbar. Removal of cell debris was performed by centrifugation (10,000× *g,* 10 min, 4 °C). Soluble protein and membrane fractions were separated by ultracentrifugation (293,000× *g*, 60 min, 4 °C). The whole-cell lysates, low-speed supernatants/pellets, and high-speed supernatants/pellets were kept at −20 °C for further analysis.

### 5.6. Sucrose-Gradient Centrifugation and OM Collection

A sucrose gradient centrifugation method modified from a previous study [42] was used. Briefly, swinging-bucket-type ultra clear tubes were filled with a sucrose gradient, layered as follows: 55% (*w*/*w*) (bottom, 1 mL), 50%, 45%, 40%, 35% (3 mL each), 30% (top, 3 mL). Cell envelopes were resuspended in gradient buffer (50 mM TEA, 250 mM sucrose, 1 mM EDTA, 1 mM DTT) and layered on top of the gradient. Ultracentrifugation (87,000× *g*, at 4 °C, 20 h) was performed using a Beckman SW28-Ti rotor with slow acceleration and no brake. All fractions 1–17 were sequentially taken from top to bottom of each gradient. To harvest the OMs, fractions 10–12 were pooled and diluted 3x in dilution buffer (50 mM TEA, 1 mM EDTA, 1 mM DTT). Pure OM samples were collected by additional ultracentrifugation (293,000× *g*, 60 min, 4 °C). The pellets were resuspended in resuspention buffer (50 mM TEA, 250 mM sucrose). Samples were analyzed by SDS-PAGE and Coomassie staining, silver staining or immunoblotting.

### 5.7. Heat-Modifiability Assay

Heat-modifiability assay was performed essentially as previously described [65]. Shortly, membrane samples were dissolved in SDS-PAGE loading buffer (125 mM Tris-HCl, pH 6.8, 20% glycerol, 0.02% bromophenol blue) containing various amounts of SDS and urea. To obtain semi-native conditions SDS was included to a final concentration of 0.1% (*w*/*v*), whereas 2% SDS (*w*/*v*), with or without 4 M urea, was included for denatured samples. Samples were subsequently kept at room temperature or incubated at 98 °C for 10 min prior to loading on semi-native polyacrylamide gels. These gels were prepared as for SDS-PAGE, but with no SDS added to the running and stacking gels. The gels were run in a normal buffer (25 mM Tris-HCl, 250 mM glycine, 0.1% SDS) at 15 mA whilst cooling the gel-cassette to prevent heating and stained with Coomassie Blue G250 or immunoblotting for further analysis.

### 5.8. Immunofluorescence Microscopy

*E. coli* BL21(DE3) cells co-expressing *Ct*-MOMP and Bam were fixed with 0.8% formaldehyde at 4 °C overnight. The cells were gently washed 3x with ice-cold PBST (0.05% Tween 20) and harvested by centrifugation (5000× *g,* 5 min, 4 °C). To permeabilize membranes and peptidoglycan, the cells were resuspended in PBS, pH 7.4, containing 0.1% Triton X-100, 100 µg/mL lysozyme, 5 mM EDTA and incubated at room temperature for 45 min [66]

After blocking with PBS containing 3% BSA for 1 h, cells were incubated with primary antisera (anti-MOMP, 1:1000 or anti-BamB, 1:200) for 1 h, subsequently with secondary antiserum (Alexa488 goat anti-rabbit, 1:200) 1 h at RT. Then, cells were immobilized by a thin layered 1% agarose on glass slides. The phase contrast and fluorescence images of cells were obtained by using LSM700 laser scanning confocal system from Zeiss (Florida, USA) and further processed using ImageJ software (http://rsb.info.nih.gov/ij/).

### 5.9. Statistical Analysis

Statistical analysis of *Ct*-MOMP expression was done using the GraphPad Prism software ver. 8.0. *p* < 0.05 “*” or *p* < 0.01 “**” was taken as significant. The error bars represent the standard error of mean (SEM).

## Data Availability

The data that support the findings of this study are available from the corresponding author upon reasonable request.

## Supplementary Materials

The following supporting information can be downloaded at: https://www.mdpi.com/article/10.3390/ijms23137393/s1.
