# Supplementary material for: Overexpression of the Bam Complex Improves the Production of Chlamydia trachomatis MOMP in the E. coli Outer Membrane
Source: Int J Mol Sci. 2022 Jul 2;23(13):7393. doi: 10.3390/ijms23137393 (PMC9266984; doi:10.3390/ijms23137393)
Supplement: Supplementary file 1 [file ijms-23-07393-s001.zip › MOMP figure supplimentary_revised.pdf]

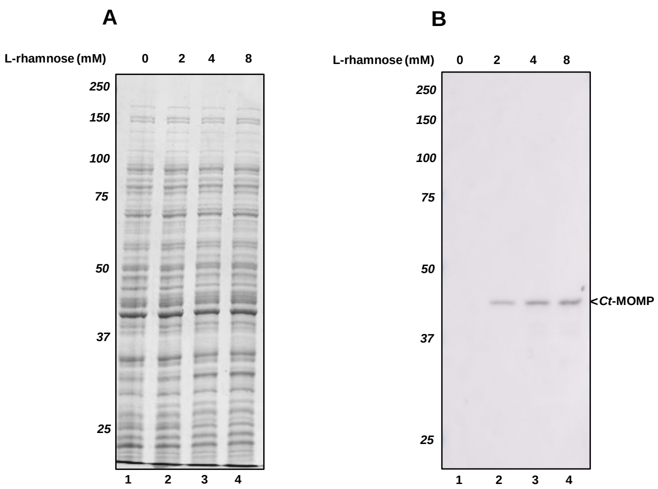

Figure S1: L-Rhamnose-induced expression of *Ct*-MOMP in *E. coli*. (A) *E. coli* BL21(DE3) whole-cell lysates expressing *Ct*-MOMP upon induction with various concentrations of L-rhamnose (2-8 mM) were analyzed by SDS-PAGE and Coomassie staining. (B) Immunoblotting of whole-cell lysates using a polyclonal antiserum against *Ct*-MOMP. Molecular weight (kDa) markers were indicated at the left side of the panels.

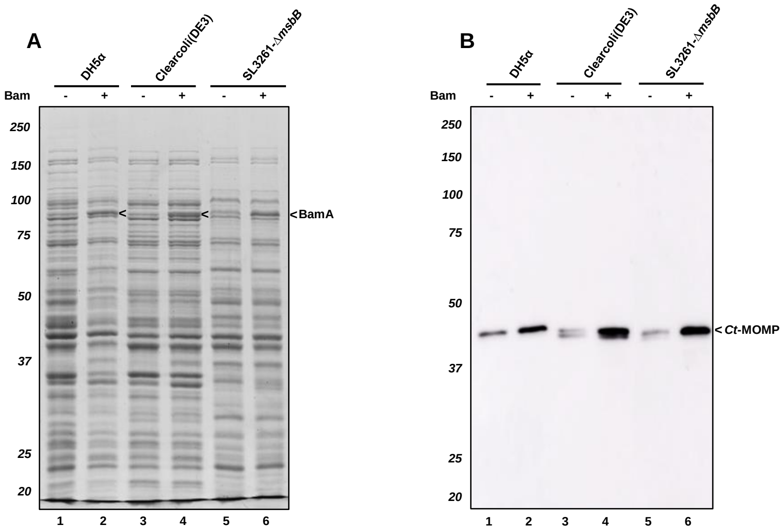

Figure S2: Improved expression of *Ct*-MOMP upon Bam co-expression in various *E. coli* and *Salmonella* strains. First, expression of the Bam complex was induced 1 h with (+) or without (-) 100 µM IPTG. Subsequently, expression of *Ct*-MOMP was induced with 8 mM L-rhamnose. Cells were grown for further 2 h, after which cells were collected. Co-overexpression of *Ct*-MOMP and the Bam complex in *E. coli* DH5α, Clearcoli(DE3), and *Salmonella* Typhimurium SL3261-Δ*msbB* analyzed by SDS-PAGE and Coomassie staining (A) or immunoblotting using the *Ct*-MOMP antiserum (B).

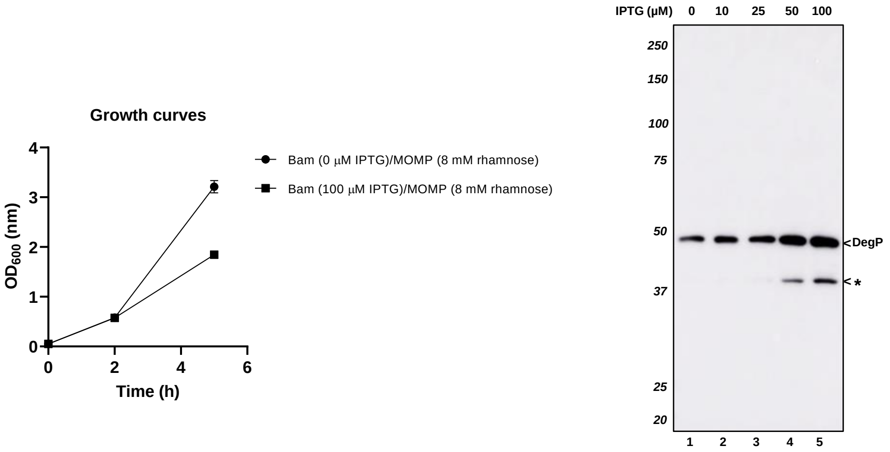

Figure S3: Co-expression of the Bam complex caused a reduction in growth (left panel) and upregulation of DegP (right panel). Growth curves of cultures were also used for Figure 1. Cells described in the legend of Figure 1 were analyzed by immunoblotting using an antiserum against DegP. An autoproteolytic fragment emerging upon upregulation of DegP was indicated (*).

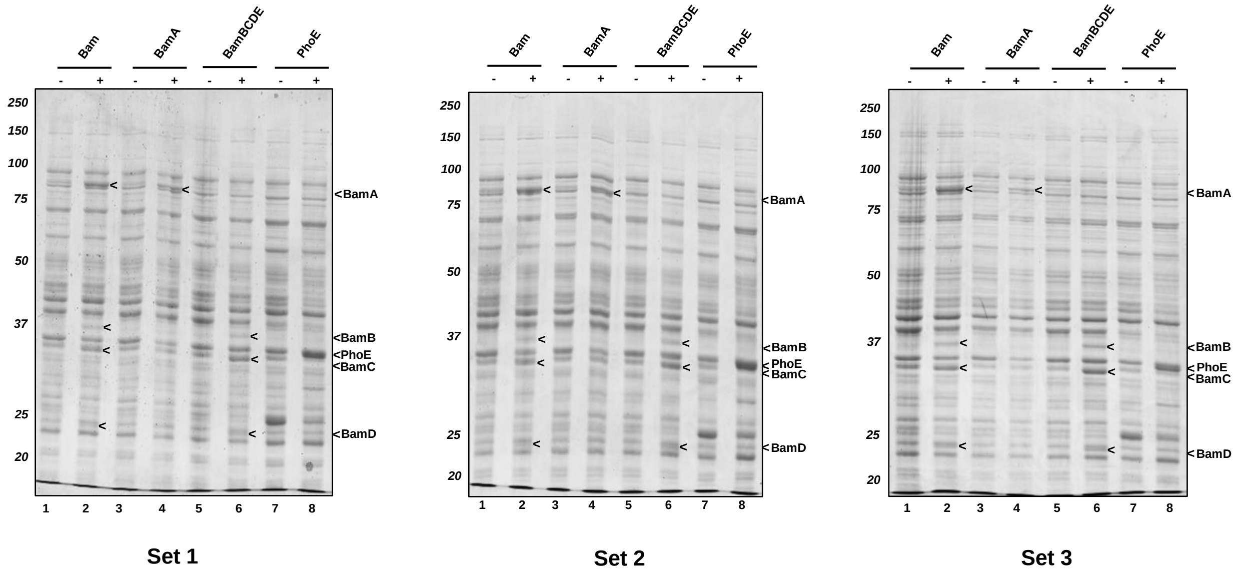

Figure S4. Coomassie-stained protein profiles of whole-cell lysates were used for quantification of *Ct*-MOMP expression in the presence of the complete Bam complex (Bam+), BamA, BamBCDE, or the OMP PhoE as the negative control. Samples from three independent experiments were analyzed (Fig. 2) by quantifying immunoblotting bands stained for *Ct*-MOMP (Figure S5) and DegP (Figure S6).

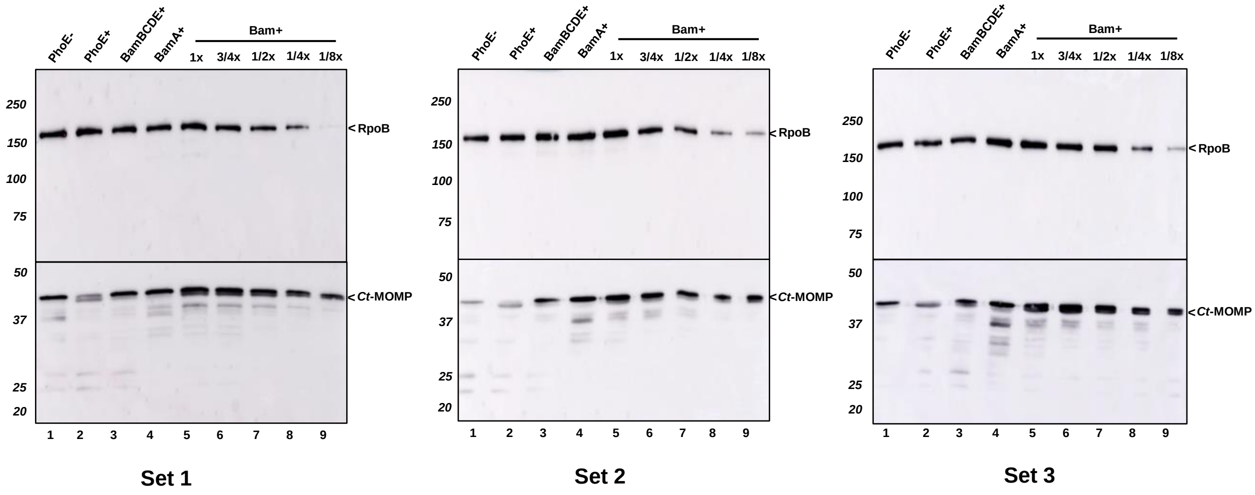

Figure S5: Immunoblotting was used for quantification of *Ct*-MOMP expression in the presence of the complete and partial Bam complex. Cells co-expressing *Ct*-MOMP and either the Bam complex (Bam+), BamA or BamBCDE, were analyzed by SDS-PAGE and immunoblotting using *Ct*-MOMP and RpoB antisera. Cells co-expressing *Ct*-MOMP induced or not induced for expression of PhoE (PhoE+ and PhoE-, respectively) were analyzed as negative controls (lane 1, 2). Samples from three independent experiments were analyzed. Signal intensities in a dilution range (1x, 3/4x, 1/2x, 1/4x, and 1/8x) of cells co-expressing the full Bam complex (Bam+) were quantified by ImageJ software (http://rsb.info.nih.gov/ij/) and used to make standard curves for *Ct*-MOMP and RpoB levels. *Ct*-MOMP and RpoB signals were then quantified based on these standard curves (see Supplementary Document 2). To compensate for loading errors, quantification of *Ct*-MOMP levels was normalized for RpoB levels quantified in the same sample. Quantifications were displayed in Figure 2A.

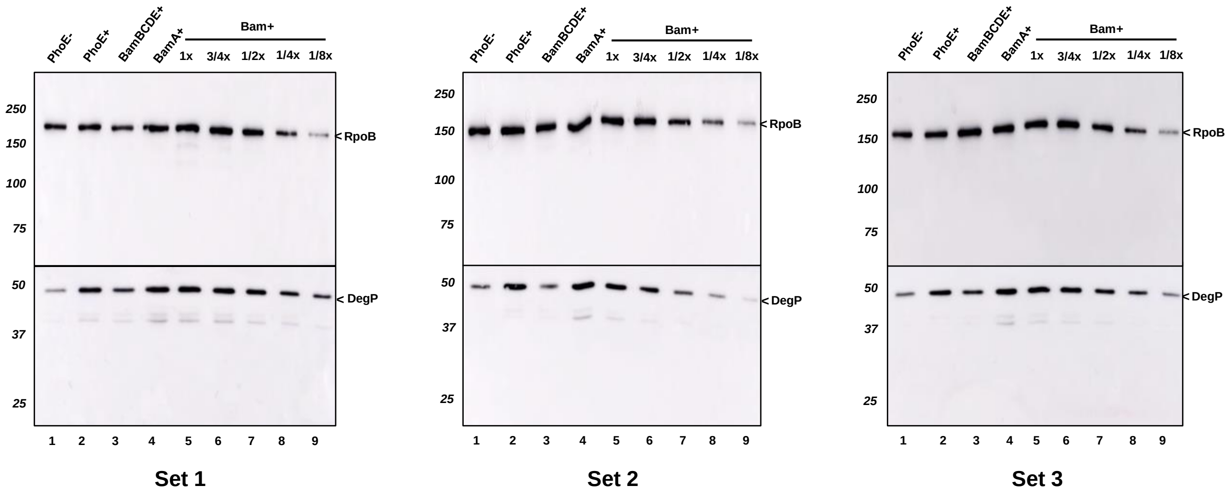

Figure S6: Immunoblottings used for quantification of DegP expression in the presence of the complete and partial Bam complex. Cells co-expressing *Ct*-MOMP and either the Bam complex (Bam+), BamA, or BamBCDE were analyzed by SDS-PAGE and immunoblotting using DegP and RpoB antisera. Cells co-expressing *Ct*-MOMP induced or not induced for expression of PhoE (PhoE+ and PhoE-, respectively) were analyzed as negative controls (lane 1, 2). Samples from three independent experiments were analyzed. Signal intensities in a dilution range (1x, 3/4x, 1/2x, 1/4x, and 1/8x) of cells co-expressing the full Bam complex (Bam+) were quantified by ImageJ software (http://rsb.info.nih.gov/ij/) and used to make standard curves for DegP and RpoB levels. DegP and RpoB signals were quantified based on these standard curves (see Supplementary Document 3). To compensate for loading errors, quantification of DegP levels was normalized for RpoB levels quantified in the same sample. Quantifications are displayed in Figure 2B.

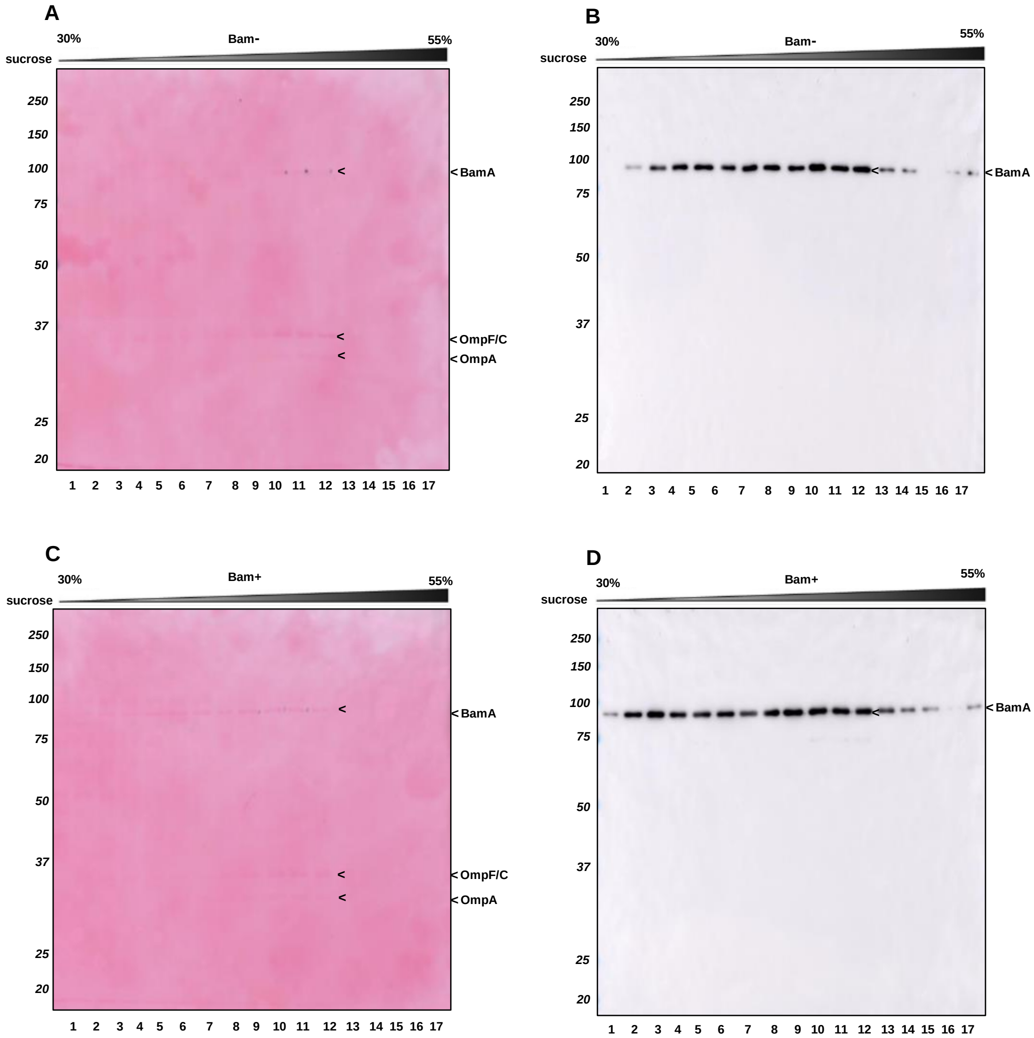

Figure S7: Localization of BamA in the OM. Bam- sucrose gradient samples described in the legend in Figure 4A were analyzed by SDS-PAGE and Ponceaus Staining (A) or immunoblotting using antiserum against BamA (B). Bam+ sucrose gradient samples described in the legend in Figure 4A were analyzed by SDS-PAGE and Ponceaus Staining (C) or immunoblotting using antiserum against BamA (D).

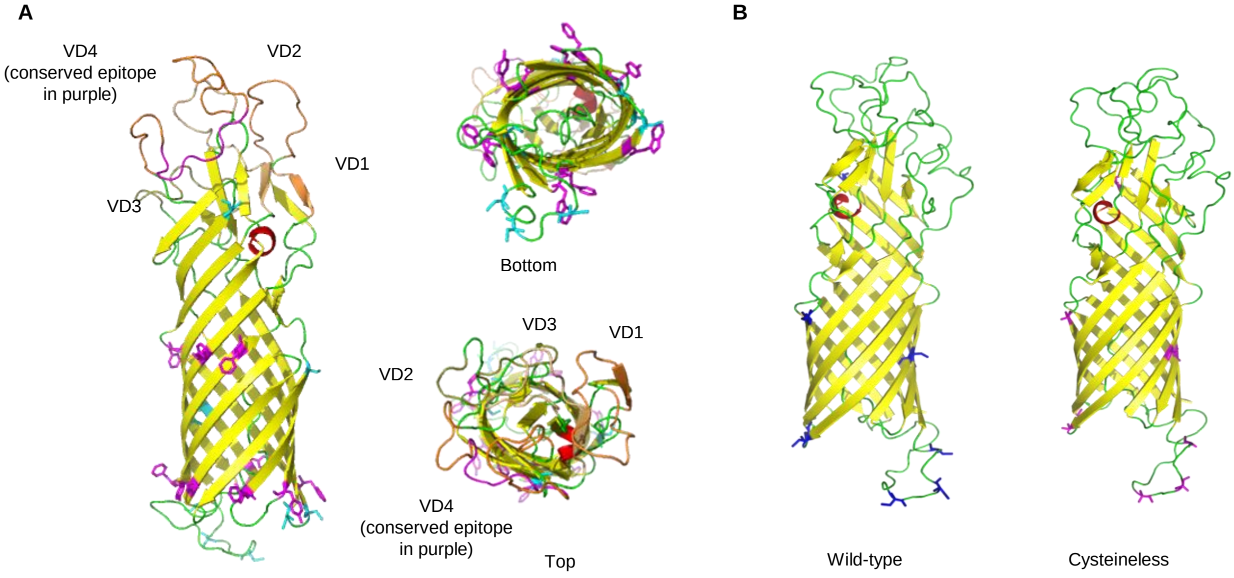

Figure S8: Three-dimensional structural models of *Ct*-MOMP. Predictions were generated using the AlphaFold algorithm with the sequence of *Ct*-MOMP of serovar D (UniProtKB-Q9RB77). (A) Cartoon representation of *Ct*-MOMP with the variable domains VDs 1-4 was indicated by different colours. The aromatic residues that line the lipid headgroups of the OM indicated in pink represent a side-view and views from the surface and periplasmic side. (B) Cartoon representation of the model obtained for wild-type *Ct*-MOMP (UniProtKB-Q9RB77) and the model obtained for the *Ct*-MOMP that has 9 cysteine residues replaced for alanines (see Supplementary Document 1). The cysteines in the wild-type model were indicated in blue (left), the alanines for the mutant model in purple (right). Models were made using Pymol (https://www.pymol.org/pymol.html?).
