# Supplementary material for: Overexpression of the Bam Complex Improves the Production of Chlamydia trachomatis MOMP in the E. coli Outer Membrane
Source: Int J Mol Sci. 2022 Jul 2;23(13):7393. doi: 10.3390/ijms23137393 (PMC9266984; doi:10.3390/ijms23137393)
Supplement: Supplementary file 1 [file ijms-23-07393-s001.zip › Supplementary Document 1.pdf]

Supplementary document 1: Amino acid sequence of *Ct*-MOMP construct used in the study

MRAKLLGIVLTTPIAISSFASTETLPVGNPAEPSLMIDGILWEGFGGDPADPAATWADAISMRV
GYYGDFVFDRVLKTDVNKEFQMGAKPTTDTGNSAAPSTLTARENPAYGRHMQDAEMFTNA
AAMALNIWDRFDVFATLGATSGYLKGNSASFNLVGLFGDNENQKTVKAESVPNMSFDQSV
VELYTDTTFAWSVGARAALWEAGAATLGASFQYAQSKPKVEELNVLANAAEFTINKPKGY
VGKEFPLDLTAGTDAATGTKDASIDYHEWQASLALSYRLNMFTPYIGVKWSRASFDADTIRI
AQPKSATAIFDTTTLNPTIAGAGDVKTGAEGQLGDTMQIVSLQLNKMKSRKSAGIAVGTTIV
DADKYAVTVETRLIDERAAHVNAQFRF

E. coli signal peptide ssOmpT
E. coli OmpT-derived linker sequence to preserve signal peptide processing
C. trachomatis serovar D (UW3/Cx) MOMP mature domain (UniProtKB Q46409: aa 22 – 393)
Cysteine to alanine substitutions

Table

Table S1. Primers used in this study

No.	Primers	Sequence (5'-3')
1	*EcoR*I/OmpT-F	AATTCAGGAGGAATTCACCATGCGGGCGAAACTTCTGG
2	*Nde*I/MOMP-R	GCAGCCTAGGGCATATGTTAAAAGCGAAACTGTGCATTAAC
3	*NdeI*-BamA-F	ACAGGAAACAGCATATGGTTAG
4	BamA-*XbaI*-R	GCAGGTCGACTCTAGAGGATCTTACCAGGTTTTACCGATG
5	*NdeI*-BamB-F	CAGGAAACAGCATATGTGGGATCTGAGAGGGACC
6	BamE-*XbaI*-R	GCAGGTCGACTCTAGAGGATC
